# Draft Genome Sequence of *Bacteroidales* Strain 6E, Isolated from a Rice Paddy Field in Japan

**DOI:** 10.1128/genomeA.01167-15

**Published:** 2015-10-08

**Authors:** Dieter M. Tourlousse, Takuya Honda, Norihisa Matsuura, Akiko Ohashi, Akio Tonouchi, Yuji Sekiguchi

**Affiliations:** a Biomedical Research Institute, National Institute of Advanced Industrial Science and Technology (AIST), Tsukuba, Ibaraki, Japan; b Faculty of Agriculture and Life Science, Hirosaki University, Hirosaki, Aomori, Japan

## Abstract

We generated a high-quality draft genome sequence of *Bacteroidales* strain 6E, a strict anaerobe newly isolated from Japanese rice paddy field soil. The genome consists of 61 contigs, with a total size of 4,436,542 bp and mean G+C content of 45.4%. Annotation predicted 3,620 protein-coding and 54 RNA genes.

## GENOME ANNOUNCEMENT

Members of the order *Bacteroidales* are prevalent in a range of anoxic/anaerobic environments rich in organic carbon (1), suggesting that they represent key populations in such ecosystems. Currently, a large number of *Bacteroidales* have not yet been cultured in the laboratory, including lineages with family-level novelty within the Greengenes database (May 2013 release [2]). We therefore undertook a study aimed at isolating novel *Bacteroidales* from rice paddy field soil. Bulk soil in paddy fields is anoxic when flooded, and degradation of organic matter is performed by a complex assemblage of bacteria (3), many of which are yet-to-be cultured and characterized. Here, we describe a high-quality draft genome sequence of a novel isolate, designated strain 6E, from soil of a rice paddy field in Aomori, Japan.

Strain 6E is a strict anaerobe capable of fermentatively utilizing various carbohydrates, including d-glucose, d-xylose, l-rhamnose, and d-cellobiose. Phylogenetic analysis based on its 16S rRNA gene sequence (DDBJ/EMBL/GenBank accession no. AB623231) suggested that strain 6E represents a novel family in the *Bacteroidales* order. Sequence similarities with other *Bacteroidales* species were <89%, and its closest cultured relative (88.8% similarity) was identified as the nitrogen-fixing species *Mangrovibacterium diazotrophicum* (accession no. JX983191).

Determination of the draft genome sequence was performed as follows. Genomic DNA was extracted from a culture of strain 6E, and Nextera XT paired-end (300 to 1,000 bp) and Nextera mate-pair libraries (1.5 to 14 kbp) were prepared from the extracted DNA. Libraries were pooled and sequenced on an Illumina MiSeq instrument with V2 chemistry (2 × 250-bp reads) at an expected coverage of >50× and >10× per genome for the paired-end and mate-pair libraries, respectively. Raw sequence reads were merged with SeqPrep with concurrent removal of sequencing adaptors, followed by quality filtering and trimming of the unmerged reads with Nesoni version 0.112 . Both merged and processed unmerged reads were combined for assembly using SPAdes version 2.5.0 (4), followed by manual improvement of the assembly, as described previously (5). Annotation was performed with the Integrated Microbial Genomes pipeline (6).

The final high-quality draft genome assembly consists of 61 contigs in 25 scaffolds and has a read coverage of 60×. The largest and *N*_50_ contig sizes are 502,414 bp and 158,264 bp, respectively. The total assembly size is 4,436,542 bp, which is comparable to the genome sizes of other *Bacteroidetes* (3.8 ± 2.0 Mbp [5]); the mean G+C content is 45.4%. Screening for *Bacteroidetes*-specific marker genes using Phyla-AMPHORA (7) indicated that the genome is practically complete, with all 215 markers being identified. The genome was predicted to contain 3,620 protein-coding genes, of which 74.1% were assigned functions. The genome further contains 54 RNA genes, including a complete set of rRNA genes. The availability of the genome sequence of strain 6E contributes to expanding our knowledge on the metabolic potential and putative roles of *Bacteroidales* in different habitats.

### Nucleotide sequence accession numbers.

This whole-genome shotgun project has been deposited in the DDBJ/EMBL/GenBank database under the accession no. BBZD00000000. The version described in this paper is BBZD01000000.
